# DCTPP1 Expression as a Predictor of Chemotherapy Response in Luminal A Breast Cancer Patients

**DOI:** 10.3390/biomedicines12081732

**Published:** 2024-08-02

**Authors:** Juan P. Muñoz, Diego Soto-Jiménez, Gloria M. Calaf

**Affiliations:** 1 Laboratorio de Bioquímica, Departamento de Química, Facultad de Ciencias, Universidad de Tarapacá, Arica 1000007, Chile; 2 Instituto de Alta Investigación, Universidad de Tarapacá, Arica 1000000, Chile

**Keywords:** breast cancer, luminal A, DCTPP1, chemoresistance, prognosis

## Abstract

Breast cancer (BRCA) remains a significant global health challenge due to its prevalence and lethality, exacerbated by the development of resistance to conventional therapies. Therefore, understanding the molecular mechanisms underpinning chemoresistance is crucial for improving therapeutic outcomes. Human deoxycytidine triphosphate pyrophosphatase 1 (DCTPP1) has emerged as a key player in various cancers, including BRCA. DCTPP1, involved in nucleotide metabolism and maintenance of genomic stability, has been linked to cancer cell proliferation, survival, and drug resistance. This study evaluates the role of DCTPP1 in BRCA prognosis and chemotherapy response. Data from the Cancer Genome Atlas Program (TCGA), Genotype-Tissue Expression (GTEx), and Gene Expression Omnibus (GEO) repositories, analyzed using GEPIA and Kaplan–Meier Plotter, indicate that high DCTPP1 expression correlates with poorer overall survival and increased resistance to chemotherapy in BRCA patients. Further analysis reveals that *DCTPP1* gene expression is up-regulated in non-responders to chemotherapy, particularly in estrogen receptor (ER)-positive, luminal A subtype patients, with significant predictive power. Additionally, in vitro studies show that *DCTPP1* gene expression increases in response to 5-fluorouracil and doxorubicin treatments in luminal A BRCA cell lines, suggesting a hypothetical role in chemoresistance. These findings highlight DCTPP1 as a potential biomarker for predicting chemotherapy response and as a therapeutic target to enhance chemotherapy efficacy in BRCA patients.

## 1. Introduction

Breast cancer (BRCA) remains a significant global health challenge. Despite advances in detection and treatment, it remains the most commonly diagnosed cancer worldwide, with an estimated 2.3 million cases and 670,000 deaths recorded in 2022 [1,2]. Additionally, it is the leading cause of cancer-related mortality among women [3]. The low survival rate and high mortality of BRCA are attributable to several factors, including late diagnosis, poor response to therapies, and a high incidence of drug resistance [4]. Therefore, understanding the molecular mechanisms underlying chemoresistance is crucial for improving therapeutic outcomes and patient survival rates.

Luminal A BRCA is the most prevalent subtype, accounting for about half of all BRCA cases [5]. Characterized by estrogen receptor (ER)-positive, progesterone receptor (PR)-positive, and human epidermal growth factor receptor 2 (HER2)-negative status, it typically exhibits low cell proliferation. Luminal A BRCA patients generally have a favorable prognosis with a low relapse rate [6,7]. While bone metastases are common in cases of recurrence, survival post-relapse tends to be longer compared to other subtypes [6].

Human deoxycytidine triphosphate pyrophosphatase 1 (DCTPP1) is an enzyme that catalyzes the hydrolysis of deoxycytidine triphosphate (dCTP) into deoxycytidine monophosphate (dCMP) and pyrophosphate [8]. This process helps maintain a balanced cellular pool of dCTP, thereby ensuring genomic stability. Additionally, DCTPP1 metabolizes non-canonical deoxycytidine analogues, such as 5-iodo-2’-deoxycytidine and 5-methyl-2’-deoxycytidine, further preserving the accuracy of DNA replication by cleaving harmful nucleotides [9,10]. DCTPP1 is localized in the nucleus, cytosol, and mitochondria of highly proliferating cells, including those in the ovary, kidney, liver, and testis. Additionally, it is overexpressed in embryonic tissue [11] and cancer stem cells [12].

Several studies have reported that DCTPP1 is overexpressed in various cancers, emphasizing its role as an oncogene. For instance, a study by Niu et al. found that DCTPP1 promotes BRCA cell proliferation via the DNA repair signaling pathway [11]. Similarly, Lu et al. demonstrated that DCTPP1 overexpression promotes tumor progression and predicts poor clinical outcome in prostate cancer [13]. In ovarian cancer, DCTPP1 has been shown to mitigate the cytotoxic effects of cisplatin by reducing reactive oxygen species (ROS) accumulation, thereby protecting cancer cells from oxidative stress-induced apoptosis [14]. Therefore, the role of DCTPP1 in cancer progression and drug resistance is particularly noteworthy.

Chemotherapy, especially with agents such as anthracyclines and taxanes, is a fundamental component of BRCA treatment [15]. It is administered preoperatively to reduce tumor size or post-operatively to eradicate any remaining tumor cells following surgery [16]. However, the efficacy of chemotherapy is often limited by the emergence of drug resistance [17]. This resistance can be intrinsic or acquired and involves various mechanisms, including enhanced DNA repair, drug efflux, and alterations in drug targets [18]. Therefore, understanding these mechanisms is essential for developing strategies to overcome resistance and improve patient outcomes [19].

Recent research has suggested that DCTPP1 may play a critical role in mediating chemotherapy resistance in cancer [14]. Overexpression of DCTPP1 has been linked to enhanced DNA repair capabilities, which can allow cancer cells to survive chemotherapy-induced DNA damage [20]. Additionally, DCTPP1’s role in nucleotide metabolism and maintenance of genomic stability further supports its involvement in chemoresistance.

In this study, we aim to investigate the role of DCTPP1 overexpression in BRCA prognosis and its impact on the response to chemotherapy. Our hypothesis is that elevated DCTPP1 levels are associated with a poor prognosis in BRCA patients undergoing chemotherapy. To test this hypothesis, we analyzed *DCTPP1* gene expression in BRCA tissues through public databases and assessed its correlation with clinical outcomes. In addition, we evaluated *DCTPP1* gene expression in cell lines subjected to common chemotherapeutic agents to confirm patient-derived data.

## 2. Methods

### 2.1. Cell Culture

Human BRCA cell lines MCF7 (#C0006008), T47D (#C0006001), and MDA-MB-231 (#C0006002) were purchased from Addexbio (San Diego, CA, USA). The MCF7 cells were cultured in Dulbecco’s Modified Eagle’s Medium (DMEM) (Gibco, Carlsbad, CA, USA) supplemented with 10 µg/mL insulin (Santa Cruz Biotechnology Inc., Santa Cruz, CA, USA) and 10% (*v*/*v*) fetal bovine serum (FBS) (Hyclone, Fremont, CA, USA). T47D cells were maintained in Roswell Park Memorial Institute (RPMI)-1640 medium (Hyclone, Logan, UT, USA) with 10% FBS. MDA-MB231 cells were grown in high glucose DMEM (4500 mg/L glucose) (Gibco, Carlsbad, CA, USA) containing 10% (*v*/*v*) FBS (Hyclone, Fremont, CA, USA). All cell lines were cultured in 75 cm^2^ flasks (Corning, Tewksbury, MA, USA) at 37 °C in a humidified atmosphere with 5% CO_2_. When cell cultures reached 80% confluence, cells were passaged using a 0.05% trypsin–EDTA solution at 37 °C for 5 min.

### 2.2. Real-Time Quantitative Reverse Transcription PCR (RT-qPCR)

Total RNA was isolated from the cell cultures using TRIzol Reagent (Invitrogen, Waltham, MA, USA) according to the manufacturer’s recommended protocol. RNA quantification was performed with the Qubit RNA BR assay kit and the Qubit 4 fluorometer (Thermo Fisher, Waltham, MA, USA). The AffinityScript qPCR cDNA Synthesis Kit (Agilent Technologies, Inc., Santa Clara, CA, USA) was used to synthesize cDNA from 1 µg of total RNA, following the manufacturer’s protocol. The reaction mixture for cDNA synthesis, with a total volume of 20 µL, included 10 µL of 2× master mix, 0.1 µg/µL of Oligo dT, and 1.0 µL of AffinityScript RT/RNase Block enzyme mix. The mixture was incubated at 37 °C for 30 min. For real-time PCR quantification, reaction mixtures were performed with a final volume of 25 µL, comprising 12.5 µL of Brilliant II SYBR Green q-RT-PCR 1-Step Master Mix (Agilent Technologies, Inc., Santa Clara, CA, USA), 0.5 µL of each primer (400 nM), 10.5 µL of nuclease-free water, and 1 µL of template cDNA. The primers set used were *β-actin* forward: TGCCGACAGGATGCAGAAG; *β-actin* reverse: GCCGATCCACACGGAGTACT; *DCTPP1* forward: CGCCTCCATGCTGAGTTTG; and *DCTPP1* reverse: CCAGGTTCCCCATCGGTTTTC. The reactions were performed in a CFX96 real-time system (Bio-Rad Laboratories, Inc., Hercules, CA, USA) under the following conditions: 94 °C for 30 s, 58 °C for 20 s, and 72 °C for 20 s for a total of 40 cycles. Each q-RT-PCR experiment was conducted in triplicate. The expression levels were normalized using *β-Actin* mRNA as a reference gene. The 2^−ΔΔCt^ method was employed to calculate the fold change in gene expression compared to control values. Melting curve analyses were performed to confirm the specificity of the PCR amplification.

### 2.3. Database Analysis

In this study, the TIMER2.0 [21,22] (http://timer.cistrome.org/, accessed on 10 June 2024) and TNM (https://tnmplot.com/analysis/, accessed on 10 June 2024) resources were utilized to explore DCTPP1 mRNA expression across various cancers and their respective normal tissues. This exploration utilized original datasets from The Cancer Genome Atlas (TCGA), the Gene Expression Omnibus (GEO) and The Genotype-Tissue Expression (GTEx) projects [23,24]. To analyze protein expression levels, we utilized the University of Alabama at Birmingham Cancer Data Analysis Portal (UALCAN, http://ualcan.path.uab.edu/, accessed on 10 June 2024) [25,26], drawing on data sourced from the Clinical Proteomic Tumor Analysis Consortium (CPTAC). The data from Human Protein Atlas (HPA) [27,28,29] (https://www.proteinatlas.org/, accessed on 10 June 2024) were used to gain insights into the immunohistochemistry of DCTPP1 in both malignant and healthy tissues.

### 2.4. Mutation Character Analysis

The cBioPortal database [30,31,32] (http://www.cbioportal.org/ accessed on 13 June 2024) was used to analyze the mutation characteristics of DCTPP1 in pan-cancers. The “TCGA Pan-Cancer Atlas Studies” cohort was selected, and “DCTPP1” was entered in the “Query” module to identify the alteration sites, types, and numbers of DCTPP1 mutations in the “Cancer Type Summary” and “Mutation” modules. 

### 2.5. Survival Data Analysis

By using the GEPIA2.0 web tool [33] (http://gepia2.cancer-pku.cn/#index, accessed on 17 June 2024), we obtained the survival data for the cancer patients with differentially expressed *DCTPP1*, including overall survival (OS) and disease-free survival (DFS). The cutoff low (50%) and cutoff high (50%) were used as the threshold values to split the lowly expressed and highly expressed groups. The statistical differences were assessed via the log-rank test. The data were compared with the publicly available Kaplan–Meier Plotter [34], PrognoScan [35] and ROC Plotter [36] web tools, available at https://kmplot.com/analysis/, http://dna00.bio.kyutech.ac.jp/PrognoScan/, accessed on 14 June 2024 and https://rocplot.com/, respectively (accessed on 20 June 2024).

### 2.6. Statistical Analysis

Statistical analysis and graphs were designed using GraphPad Prism version 5.0 software (GraphPad Software, Inc., La Jolla, CA, USA). The Student’s *t*-test was used to compare the expression of DCTPP1 between tumor tissues and corresponding normal tissues. Spearman’s rank correlation test was used to analyze the correlations between DCTPP1 expression and patient survival, as this is a non-parametric test appropriate for analyzing correlations between two non-normally distributed variables. The statistical significance of the Kaplan–Meier survival curves was assessed using the log-rank test, which is suitable for comparing survival distributions between two groups. *p* < 0.05 was considered to indicate a statistically significant difference, whereby *, *p* < 0.05; **, *p* < 0.01; ***, *p* < 0.001; ****, *p* < 0.0001.

## 3. Results

### 3.1. DCTPP1 Expression Levels and Alteration Frequency in Pan-Cancers

To investigate the role of DCTPP1 in human tumors, we used publicly available gene expression databases to compare mRNA transcript abundance between tumor and matched normal tissue specimens. The data provided by TIMER2.0 indicated a notable up-regulation of *DCTPP1* in several types of carcinomas (Figure S1A). To validate these results, we turned to the TNMplot resource to explore *DCTPP1* expression within the TCGA and GTEx datasets (Figure S1B). This web tool corroborated the up-regulation of *DCTPP1* in a majority of the cancers highlighted by TIMER2.0.

**Figure 1:**
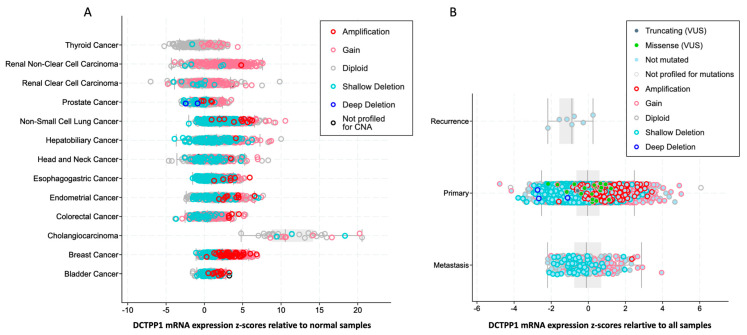
*DCTPP1* alteration frequency in pan-cancers. A) Distribution of *DCTPP1* mRNA expression z−scores relative to normal samples across multiple cancer types. The z-scores are shown for various genomic alterations including amplification, gain, diploid, shallow deletion, and deep deletion. B) *DCTPP1* mRNA expression z-scores relative to all samples in different stages of cancer. The z−scores are shown for various mutation types such as truncating (VUS), missense (VUS), not mutated, and samples not profiled for mutations, as well as genomic alterations including amplification, gain, diploid, shallow deletion, and deep deletion.

To further elucidate the DCTPP1 up-regulation in several cancer types, we examined its genetic alteration frequency across different tumors using data from the TCGA database, retrieved via cBioPortal. The results of the analysis depicted in Figure 1 showed that significant alterations were observed in the majority of cancers that display DCTPP1 overexpression. In these cases, the predominant alterations were amplifications and gains, with fewer instances of shallow and deep deletions (Figure 1A). Interestingly, we observed significant up-regulation and genetic alteration of *DCTPP1* in BRCA. Specifically, BRCA cases exhibited a high frequency of *DCTPP1* amplifications and gains, which could explain the overexpression depicted in Figure 1. Additionally, we analyzed the alteration frequency of different stages of cancer progression, including primary, recurrent, and metastatic tumors. The primary tumors showed the highest frequency of *DCTPP1* alterations, predominantly amplifications and gains, while recurrent and metastatic tumors displayed fewer alterations (Figure 1B). In summary, these analyses demonstrated a notable up-regulation of *DCTPP1* in several types of carcinomas. Additionally, we observed significant genetic alterations in *DCTPP1* in BRCA, predominantly amplifications and gains.

### 3.2. DCTPP1 Is Up-Regulated across Tumor Grades and Cancer Stages in BRCA

**Figure 2:**
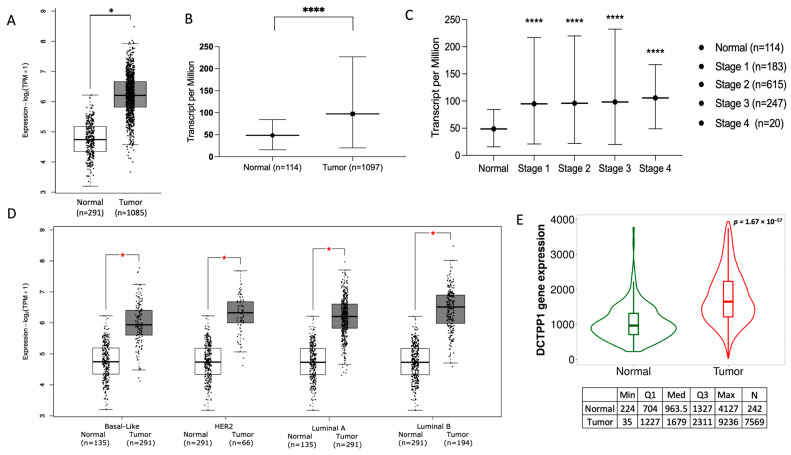
Comparative analysis of DCTPP1 gene expression across tumor stages and BRCA subtypes. (**A**) Comparison of DCTPP1 expression in normal (n = 291) and tumor (n = 1085) tissues. (**B**) Transcript per million (TPM) comparison between normal (n = 114) and tumor (n = 1097) tissues. (**C**) DCTPP1 expression across different BRCA stages: stage 1 (n = 183), stage 2 (n = 615), stage 3 (n = 247), and stage 4 (n = 20), with normal tissue (n = 114) as a reference. (**D**) DCTPP1 expression in different BRCA subtypes: basal-like, HER2, luminal A, and luminal B, with corresponding normal tissues. Data retrieved from a GEPEIA (**E**) violin plot showing DCTPP1 gene expression in normal (n = 242) and tumor (n = 7569) tissues. (* *p* < 0.05; **** *p* < 0.0001).

Given the high rates of *DCTPP1* gene amplifications observed in BRCA, we analyzed its mRNA expression levels across tumor grades and cancer stages in BRCA cases. In accordance with RNA sequence data provided by Gepeia2.0 and UALCAN web tools, we observed statically significant *DCTPP1* overexpression in BRCA tumors compared with normal tissue (Figure 2A,B), across its different stages (Figure 2C) and subtypes (Figure 2D).

To strengthen these data, we further investigated publicly available transcriptome-wide datasets hosted on the TNM platform. This analysis corroborated the significant up-regulation of *DCTPP1* mRNA in tumor tissues compared to controls (*p*-value = 1.67 × 10^−57^). The median expression level in tumors (1679) was markedly higher than that in normal tissues (963.5), providing further evidence supporting our initial observations. This overexpression correlated with advanced tumor grades and stages, indicating its potential role in tumor progression and aggressiveness (Figure 2E). These data show that *DCTPP1* could be probed as a reliable biomarker, with high specificity at elevated cut-off levels, underscoring its diagnostic and prognostic value in BRCA.

**Figure 3:**
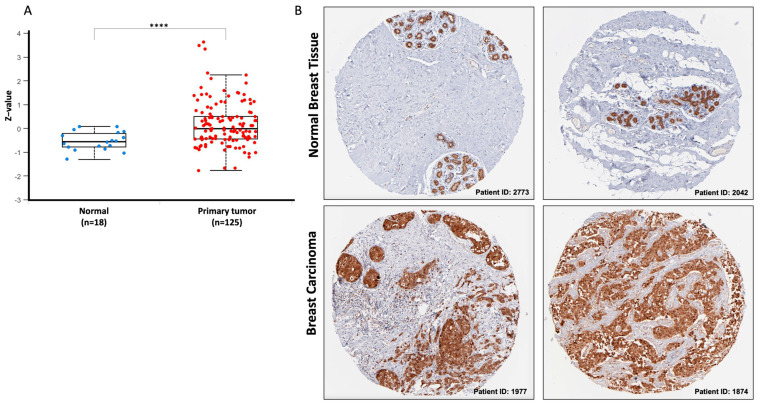
DCTPP1 protein expression between normal and BRCA tissues. (**A**) Box plot illustrating DCTPP1 protein expression levels in 18 normal breast tissue samples and 125 BRCA samples, analyzed using the UALCAN web tool. (**B**) Immunohistochemical analysis of DCTPP1 protein levels in BRCA samples from the HPA repository. **** *p* < 0.0001.

To corroborate the findings of the transcriptomic data, we examined the protein expression level of DCTPP1 in 125 BRCA samples and 18 normal samples using the UALCAN web tool (Figure 3A). This analysis revealed a statistically significant difference in DCTPP1 protein levels between BRCA samples and their normal counterparts (*p* = 6.6 × 10^−50^). This finding confirms that the increase in *DCTPP1* mRNA observed in BRCA cases is also accompanied by an increase in protein levels. In addition, immunohistochemical analysis using data from the HPA repository provided further evidence. Normal breast tissues showed cytoplasmic staining, indicating DCTPP1 protein expression. By contrast, BRCA tissues exhibited significantly higher levels of DCTPP1, with a tenfold increase in staining intensity compared to normal tissues. This staining was used to visually demonstrate the differential expression levels of DCTPP1, underscoring its up-regulation during the oncogenic transformation of breast tissue (Figure 3B). These findings support the hypothesis that DCTPP1 expression is increased during the oncogenic transformation of breast tissue.

Collectively, our analyses reveal that DCTPP1 is up-regulated across all tumor grades and stages in BRCA, at both mRNA and protein levels.

### 3.3. Correlation between DCTPP1 Gene Expression and Established Markers in ER-Positive, Luminal A BRCA Patients

**Figure 4:**
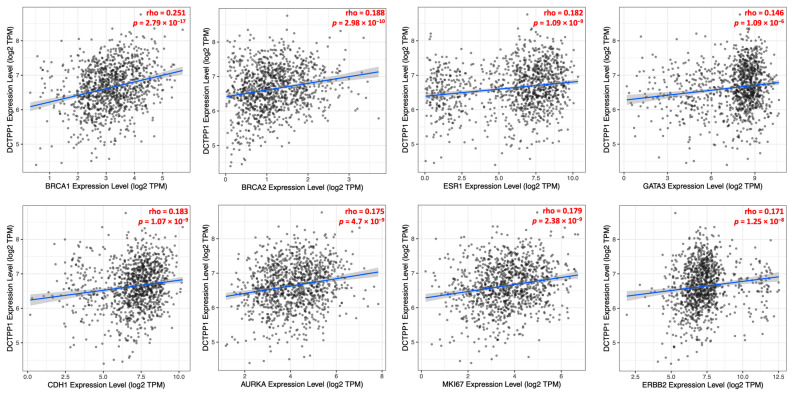
Scatter plots showing the correlation between DCTPP1 gene expression and established chemotherapy-induced markers in BRCA. The scatter plots are shown with linear regression lines and correlation coefficients (ρ) for each gene. The blue lines in each plot represent the linear regression fit, which indicates the trend or relationship between the expression levels of DCTPP1 and the respective gene. The grey shaded area around each blue regression line represents the confidence interval. This analysis was carried out using the TIMER2.0 web tool.

To investigate the association between DCTPP1 overexpression and established BRCA biomarkers, we conducted correlation analyses employing the TIMER2.0 database. Specifically, we assessed the expression levels of DCTPP1 in relation to a selected panel of genes known to be involved in the pathogenesis and prognosis of BRCA. As shown in Figure 4, a positive correlation between DCTPP1 expression and BRCA1 (ρ = 0.251, *p* = 2.79 × 10^−17^), BRCA2 (ρ = 0.188, *p* = 2.98 × 10^−10^), ESR1 (ρ = 0.175, *p* = 4.7 × 10^−9^), and GATA3 (ρ = 0.146, *p* = 1.09 × 10^−6^) was found. Furthermore, we assessed the relationship between DCTPP1 expression and key markers associated with poor prognosis in BRCA, including CDH1 (ρ = 0.183, *p* = 1.07 × 10^−9^), AURKA (ρ = 0.175, *p* = 4.7 × 10^−9^), MKI67 (ρ = 0.179, *p* = 2.38 × 10^−9^), and ERBB2 (ρ = 0.171, *p* = 1.25 × 10^−8^). The *p*-values of these correlations underscore the intertwined relationship between DCTPP1 and the key molecular drivers of BRCA. The consistent positive correlations across multiple markers highlight DCTPP1’s potential role in the molecular pathology of BRCA, suggesting its hypothetical utility in prognostic assessments.

### 3.4. DCTPP1 as a Potential Marker of Poor Prognosis in Chemotherapy-Treated BRCA Patients

**Figure 5:**
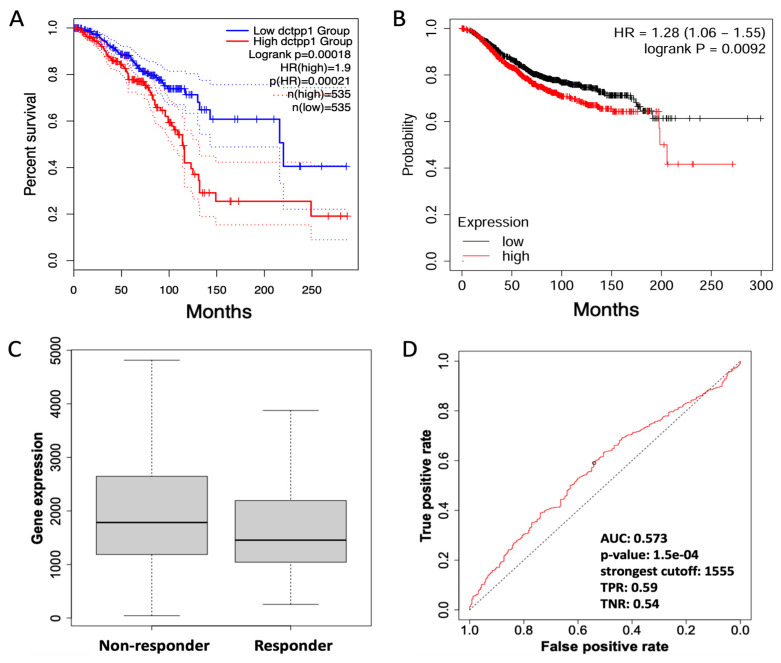
Evaluation of *DCTPP1* expression and its association with BRCA prognosis and treatment response. (**A**) Kaplan–Meier survival curves showing the OS based on *DCTPP1* expression levels in BRCA patients. Patients are grouped into high- and low-*DCTPP1* expression categories. Data obtained from GEPEIA web tool. (**B**) Kaplan–Meier plot of the OS for BRCA patients with high (red line) and low (black line) *DCTPP1* expression. The hazard ratio (HR) and log-rank *p*-value are indicated. Data obtained from Kaplan–Meier Plotter web tool. (**C**) Box plot comparing *DCTPP1* gene expression between non-responders and responders to chemotherapy in BRCA patients. (**D**) ROC curve analysis evaluating the predictive power of *DCTPP1* expression for chemotherapy response in BRCA patients, with the AUC, *p*-value, and optimal cutoff point indicated. Data retrieved from ROC Plotter web tool.

Given the positive correlations observed between DCTPP1 and markers associated with poor prognosis of BRCA, we evaluated whether DCTPP1 serves as a potential marker of poor prognosis in chemotherapy-treated BRCA patients by assessing data from TCGA, GTEx, and GEO databases using two online resources: GEPIA and Kaplan–Meier Plotter. For this analysis, patients were categorized into two groups based on their DCTPP1 expression levels: high expression and low expression. The overall survival (OS) analysis (Figure 5A,B) indicated that patients with high DCTPP1 expression levels (red line) had significantly worse survival rates compared to those with low DCTPP1 expression levels (blue and black lines). The high DCTPP1 group showed a higher hazard ratio (HR = 1.28), indicating a higher risk of mortality compared to the low DCTPP1 group (Figure 5B). This suggests that high DCTPP1 expression is associated with poorer disease-free survival in BRCA patients. Furthermore, we used the ROC Plotter web tool to evaluate the expression levels of the DCTPP1 gene in BRCA patients with a positive ER status and the luminal A subtype treated with chemotherapy [36]. The data showed that non-responders had higher mean (2004) and median (1738) DCTPP1 expression levels compared to responders, who had mean and median levels of 1569 and 1318, respectively (Figure 5C). These data suggest that non-responders to chemotherapy have higher DCTPP1 expression levels than responders. The area under the receiver operating characteristic (ROC) curve (AUC) is 0.615, indicating predictive power with a statistically significant *p*-value of 1× 10^−5^ (Figure 5D). The Mann–Whitney test also showed a significant difference between the two groups (*p*-value = 9 × 10^−5^), and the fold change was 1.3, suggesting an increase in DCTPP1 expression in non-responders.

Overall, these results suggest the potential of DCTPP1 as a biomarker for predicting chemotherapy response in ER-positive, luminal A BRCA patients. The statistical significance and consistent values across different sets of data strengthen these findings.

### 3.5. DCTPP1 Expression Levels in Luminal A BRCA Cell Lines Treated with 5-FU

**Figure 6:**
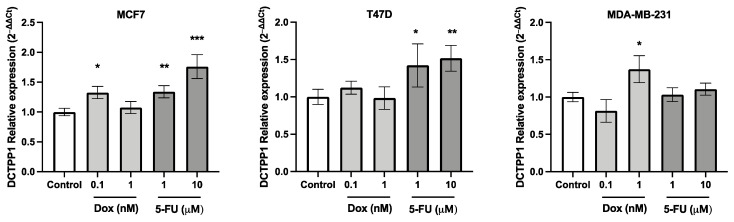
RT-qPCR analysis of *DCTPP1* gene expression levels in BRCA cell lines exposed to Doxorubicin (Dox) and 5−fluorouracil over 24 h. Bar graphs represent the relative expression of *DCTPP1* normalized to the *β-actin* gene. Data are presented as the mean ± standard error of the mean from triplicate experiments. (* *p* < 0.05; ** *p* < 0.01; *** *p* < 0.001).

To elucidate the role of DCTPP1 in modulating chemotherapy responses, we examined its expression profiles in luminal A and triple-negative BRCA cell lines subjected to 5-fluorouracil (5-FU) and doxorubicin (Dox) treatment, two widely employed chemotherapeutic agents in BRCA. Specifically, we selected the luminal A cell lines MCF7 and T47D, known for their hormone receptor-positive status, and compared them with the hormone receptor-negative cell line MDA-MB-231. This comparative approach aimed to dissect the distinct impacts of DCTPP1 across different molecular subtypes of BRCA. The cell lines were cultured and subjected to two concentrations of 5-FU and Dox over 24 h. Afterwards, RT-qPCR was utilized to quantitatively assess the levels of DCTPP1 mRNA, providing insights into its regulatory dynamics in response to chemotherapeutic treatment. The results demonstrated that DCTPP1 expression was significantly up-regulated following 5-FU treatment in the MCF7 and T47D cell lines (Figure 6). Specifically, cells exposed to higher concentrations of 5-FU exhibited a pronounced increase in DCTPP1 expression when compared to untreated controls. Conversely, in the triple-negative MDA-MB-231 cell line, DCTPP1 expression did not exhibit a statistically significant change, underscoring a distinct molecular response to this agent. The overexpression observed in MCF7 and T47D suggest that DCTPP1 may serve as a potential biomarker for predicting chemotherapy response in ER-positive, luminal A BRCA cases, highlighting its potential utility in this cancer subtype.

## 4. Discussion

BRCA remains one of the most prevalent cancers worldwide, with significant morbidity and mortality rates [37]. Luminal A is the most common BRCA subtype, constituting 50–60% of all cases. This subtype is defined by the activation of genes typically expressed in the mammary ductal epithelium and regulated by the ER transcription factor [38]. Patients with luminal A BRCA generally exhibit a favorable prognosis compared to other subtypes, with a significantly lower recurrence rate [39].

Historically, chemotherapy has been the primary treatment for BRCA, with treatment decisions guided by immunohistochemical markers and tumor characteristics [6]. Nevertheless, the emergence of chemotherapy resistance remains a critical challenge. To address this, this study investigates the potential of *DCTPP1* as a predictor of chemotherapy response in patients with luminal A BRCA. To evaluate this, the study employed a multi-faceted approach, integrating data from multiple publicly available databases (TCGA, GTEx, GEO) analyzed using bioinformatics tools such as GEPIA and Kaplan–Meier Plotter.

The results analyses revealed a significant correlation between high *DCTPP1* expression and poor overall survival in BRCA patients, with a particular focus on the luminal A subtype. The robustness of these findings was enhanced by the large sample sizes and the use of multiple validation datasets. Notably, the quantification of *DCTPP1* expression in cell lines after the exposure to chemotherapeutic compounds corroborated these findings, showing higher *DCTPP1* levels in luminal A cell lines compared to triple-negative breast cancer (TNBC). The consistent observation of DCTPP1’s association with chemotherapy response across multiple datasets strongly supports its potential as a predictive biomarker for BRCA luminal A patients. These findings further suggest DCTPP1’s involvement in the development of chemoresistance.

The survival analysis conducted using the Kaplan–Meier Plotter and GEPIA tools indicated that patients with elevated *DCTPP1* expression have significantly worse survival rates than those with lower expression. The statistical rigor of these analyses, including the use of log-rank tests for survival data, lends substantial weight to the prognostic significance of *DCTPP1*. In addition, the association of high *DCTPP1* levels with nearly double the risk of mortality in chemoresistant patients highlights its potential utility as a prognostic biomarker.

Further deepening the study’s implications, *DCTPP1* expression was analyzed in relation to other well-established prognostic markers such as *BRCA1*, *BRCA2*, and *ESR1*. The positive correlations observed suggested that DCTPP1’s role in cancer biology may be intertwined with fundamental pathways governing tumor progression and hormone receptor signaling. This aspect of the research opens up new avenues for understanding how *DCTPP1* could be integrated into existing prognostic models to improve the accuracy of BRCA prognostication.

The clinical relevance of these findings is substantial. High *DCTPP1* expression is associated with nearly double the risk of mortality, emphasizing the need for personalized treatment strategies. With the development of a predictive model based on *DCTPP1* expression, clinicians could potentially tailor chemotherapy regimens to individual patient profiles, thus optimizing therapeutic outcomes. The ROC curve analysis further emphasized the predictive power of *DCTPP1* expression, suggesting that incorporating this biomarker into clinical practice could significantly impact decision-making processes in BRCA treatment.

The in vitro component of the study involved luminal A BRCA cell lines (MCF7 and T47D) and a triple-negative BRCA cell line (MDA-MB-231). The differential expression of DCTPP1 in these cell lines in response to chemotherapy underscored the specificity of DCTPP1′s role in luminal A BRCA. The lack of significant changes in *DCTPP1* expression in the triple-negative cell line suggested that the role of DCTPP1 in chemoresistance may be subtype-specific, primarily affecting hormone receptor-positive BRCA. Several factors could contribute to this phenomenon, and while our study primarily focused on expression levels, we can propose some potential explanations that warrant further investigation: The distinct molecular characteristics and signaling pathways inherent to these subtypes could influence the regulation of DCTPP1 expression in response to 5-FU treatment. For instance, differential expression profiles of the transcriptome in MCF7 and MDA-MB-231 have been described [40], suggesting that variations in gene regulation mechanisms—such as transcription factor binding, epigenetic modifications, or non-coding RNA interactions—may contribute to the differential expression of DCTPP1. It is also plausible that post-translational modifications (PTMs) could play a role in the regulation of DCTPP1 stability, localization, or activity, thereby contributing to the observed differences in expression levels. Additionally, the differential response to 5-FU treatment could reflect variations in drug uptake, metabolism, or cellular stress responses, as has been demonstrated with other drugs [41,42]. These differences might influence cellular demand for DCTPP1’s enzymatic functions, leading to variations in its expression. To definitively determine the underlying causes of differential expression of DCTPP1, further investigations are needed. These should include studies on the transcriptional and post-transcriptional regulation of DCTPP1, as well as the potential involvement of PTMs.

The extended findings on *DCTPP1* expression furnish a robust framework for its role as a key player in mediating chemoresistance in luminal A BRCA. By demonstrating significant associations with poor therapeutic outcomes and lower survival rates, our study makes a case for the clinical evaluation of *DCTPP1* as both a predictive and prognostic biomarker. On the other hand, even though this study did not elucidate the specific mechanisms underlying DCTPP overexpression, several potential pathways through which *DCTPP1* might contribute to chemoresistance can be proposed. One possible mechanism is through enhanced DNA repair capabilities, allowing cancer cells to survive chemotherapy-induced DNA damage. Another is the maintenance of the nucleotide pool balance, preventing the incorporation of harmful nucleotides into DNA. These mechanisms underscore the multifaceted role of *DCTPP1* in cancer cell survival and resistance.

Further research is needed to elucidate the precise molecular mechanisms by which DCTPP1 contributes to chemoresistance. Investigating the interaction of DCTPP1 with other molecular pathways involved in DNA repair and nucleotide metabolism could provide deeper insights. Additionally, exploring potential inhibitors of DCTPP1 or strategies to down-regulate its expression may offer new therapeutic avenues to overcome chemoresistance.

In conclusion, this study highlights the significant role of DCTPP1 in predicting chemotherapy response in luminal A BRCA patients. The findings suggest that high *DCTPP1* gene expression is a marker of poor prognosis and chemoresistance, providing a potential target for therapeutic intervention.

## Data Availability

The results published here are based in whole or part upon original data generated by the TCGA Research Network: https://www.cancer.gov/tcga (accessed on 10 June 2024) GTEx: https://www.gtexportal.org (accessed on 10 June 2024); Human Protein Atlas: https://www.proteinatlas.org/, CPTAC: https://proteomics.cancer.gov/programs/cptac (accessed on 10 June 2024).

## Supplementary Materials

The following supporting information can be downloaded at: https://www.mdpi.com/article/10.3390/biomedicines12081732/s1. Figure S1. DCTPP1 expression increases in tumor samples.
