# Supplementary material for: DCTPP1 Expression as a Predictor of Chemotherapy Response in Luminal A Breast Cancer Patients
Source: Biomedicines. 2024 Aug 2;12(8):1732. doi: 10.3390/biomedicines12081732 (PMC11351553; doi:10.3390/biomedicines12081732)
Supplement: Supplementary file 1 [file biomedicines-12-01732-s001.zip › biomedicines-3135099-supplementary.pdf]

Supplementary figure

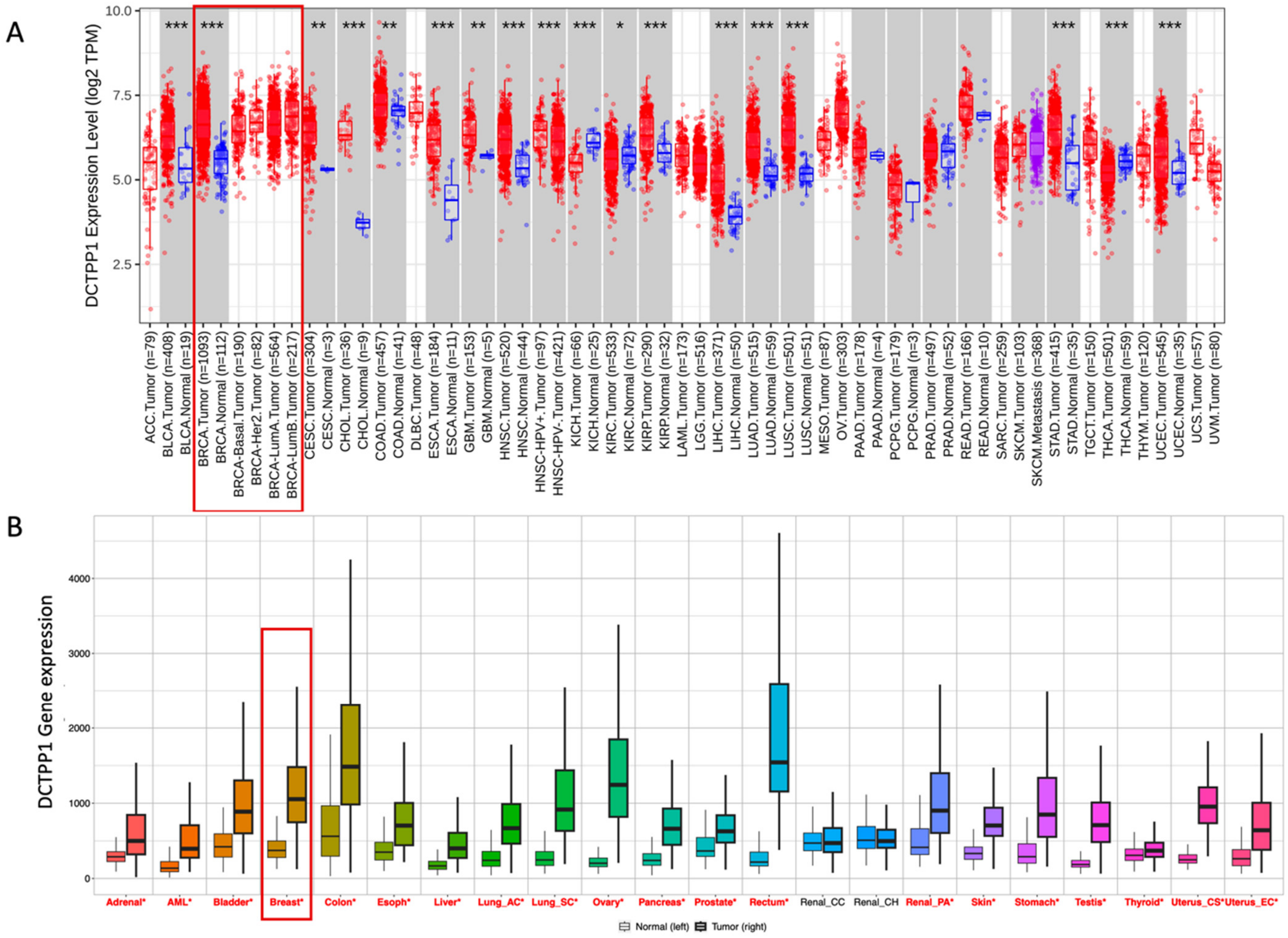

Figure S1. DCTPP1 expression increases in tumor samples. Differential expression analysis of DCTPP1 in TCGA, analyzed via TIMER2.0 (A) and TNM plot (B). Red letters indicate statistical significance. Abbreviations: BLCA (Bladder cancer), BRCA, CESC (Cervical squamous cell carcinoma and endocervical adenocarcinoma), CHOL (Cholangiocarcinoma), COAD (Colon adenocarcinoma), ESCA (Esophageal carcinoma), GBM (Glioblastoma multiforme), HNSC (Head and neck squamous cell carcinoma), KICH (Kidney chromophobe), KIRP (Kidney renal papillary cell carcinoma), LIHC (Liver hepatocellular carcinoma), LUAD (Lung adenocarcinoma), LUSC (Lung squamous cell carcinoma), PAAD (Pancreatic adenocarcinoma), PRAD (Prostate adenocarcinoma), READ (Rectum adenocarcinoma), STAD (Stomach adenocarcinoma), THCA (Thyroid carcinoma), and UCEC (Uterine corpus endometrial carcinoma).
